# A New Deep Learning Framework for Imbalance Detection of a Rotating Shaft

**DOI:** 10.3390/s23167141

**Published:** 2023-08-12

**Authors:** Muhammad Wisal, Ki-Yong Oh

**Affiliations:** Departement of Mechanical Convergence Engineering, Hanyang University, 222, Wangsimni ro, Seongdong gu, Seoul 04763, Republic of Korea

**Keywords:** unbalance detection, artificial neural network, deep learning, statistical property, Short-Time Fourier Transform, optimization

## Abstract

Rotor unbalance is the most common cause of vibration in industrial machines. The unbalance can result in efficiency losses and decreased lifetime of bearings and other components, leading to system failure and significant safety risk. Many complex analytical techniques and specific classifiers algorithms have been developed to study rotor imbalance. The classifier algorithms, though simple to use, lack the flexibility to be used efficiently for both low and high numbers of classes. Therefore, a robust multiclass prediction algorithm is needed to efficiently classify the rotor imbalance problem during runtime and avoid the problem’s escalation to failure. In this work, a new deep learning (DL) algorithm was developed for detecting the unbalance of a rotating shaft for both binary and multiclass identification. The model was developed by utilizing the depth and efficacy of ResNet and the feature extraction property of Convolutional Neural Network (CNN). The new algorithm outperforms both ResNet and CNN. Accelerometer data collected by a vibration sensor were used to train the algorithm. This time series data were preprocessed to extract important vibration signatures such as Fast Fourier Transform (FFT) and Short-Time Fourier Transform (STFT). STFT, being a feature-rich characteristic, performs better on our model. Two types of analyses were carried out: (i) balanced vs. unbalanced case detection (two output classes) and (ii) the level of unbalance detection (five output classes). The developed model gave a testing accuracy of 99.23% for the two-class classification and 95.15% for the multilevel unbalance classification. The results suggest that the proposed deep learning framework is robust for both binary and multiclass classification problems. This study provides a robust framework for detecting shaft unbalance of rotating machinery and can serve as a real-time fault detection mechanism in industrial applications.

## 1. Introduction

The predominant factor contributing to mechanical failures in rotating machinery is the presence of vibrations [1], commonly caused by unbalance in the rotor [2,3]. “*Rotor unbalance is a condition in which the Centre of mass of a rotating assembly, typically the shaft and its fixed components like disks, and blades* etc. *is not coincident with the Centre of rotation*” [3]. Frequent causes of rotor unbalance are vibrations due to some externally applied load, bent shafts, or asymmetric mass distribution [4,5]. Other causes of rotor unbalance include bearing damage [6] and casting inaccuracies like porosity, non-uniform material density, asymmetric shaft fatigue, or faults in ball bearings that support the shaft [7,8]. Vibrations due to rotor unbalance can easily escalate to the failure of critical machine components such as bearings, gears, couplings, etc. [9]. Constant health monitoring in rotating machinery is needed to avoid structural damage.

Usually, three types of machinery maintenance approaches are adopted in industry:

**Reactive maintenance**, often referred to as the “run till failure” approach;

**Preventive maintenance**, when machinery is periodically overhauled regardless of the condition of parts;

**Predictive maintenance**, which is the process of machinery health monitoring during runtime in order to predict when and which parts are likely to fail [10].

Reactive and preventive maintenance do not require any sensors to constantly monitor the condition of the machinery, whereas predictive maintenance does need health monitoring sensors like acoustic, vibration, shock pulse monitoring, wear debris, and thermal sensors [11]. Vibration sensor-based condition monitoring is common in industry as it allows for identifying 90% of faults or failures in machines [1].

In contrast to preventive and reactive maintenance, predictive maintenance is less costly and provides a constant analysis of machinery conditions [10]. Predictive-maintenance-based fault identification methods can be classified into two groups according to their dependence on the structural model: ***model-based methods and signal-based methods*** [12,13]. Model-based methods can estimate the damage location and severity by improving the mathematical model of the structure using experimental data or Finite Element Analysis data [12]. On the other hand, *signal-based methods* detect damage by comparing the structural responses before and after the damage. The method makes use of recorded signals rather than explicit input–output models for fault prediction. Damage is defined by damage indices, which are determined from the time–frequency domain analysis or by modal analysis [14,15]. Artificial Intelligence (AI), as a class of signal-based methodology, has emerged as a powerful tool for the identification of multivariable nonlinear systems.

Exploiting AI for motor fault detection has been of interest since 1993. Mo-Yuen Chow et al. [16] concluded that the size of training data directly affects the accuracy of the AI system. Bo Li et al. (1998) showed that FFT values of a vibration signal spectrum can be used as relevant features of the vibration dataset [17]. The authors of [4,16] proved that optimizing network parameters like learning rate, momentum, and neuron size is important for increasing accuracy. S. Rajakarunakaran et al. used an Adaptive Resonance Theory (ART) network as a vector classifier. It accepts time series vibration signals as the input vector and classifies them into one of the categories (clusters) depending on which of the stored patterns it most resembles [18]. The unsupervised method is good for clearly distinguished classes of balanced and highly unbalanced machine signals where the difference is high. However, the accuracy decreases fast when the unbalance factor is reduced. Chih-Hao Chen et al. (2008) proposed a new fault diagnosis procedure for rotating machinery fault detection using wavelet packet–fractal technology and a radial basis function neural network. The fault categories considered were unbalance, shaft misalignment, and looseness [19]; however, the level of unbalance was not considered. For diagnosing the type of unbalance, Sendhilkumar et al. proposed feeding vibration acceleration data features as input into Elman neural networks [20]. Weining Lu et al. [21] introduced a deep neural network for Domain Adaptation in Fault Diagnosis (DAFD), which increases classifier accuracy by adapting the neurons’ weights to new values by deploying the weight regularization term, hence strengthening the representative features of the original data. Tang et al. [22] trained an SVM model using chaotic particle swarm optimization (PSO) to classify inputs into the multiple faults of rotating machines. Similar to the references [22,23], several other authors adopted SVM in fault classification models [24,25,26,27]. However, SVM runs into target class overlapping problems when faced with noisy and industrial-level large-scale data [28].

Rotor imbalance has been studied by various researchers recently. The authors of [29] presented an online unbalance rotor fault detection technique. The method carries out statistical and frequency domain analyses of the vibration data as well as current signatures; however, unlike machine learning methods, the proposed method requires expert-level knowledge of spectral analysis. Brando et al. used SVM for multiclass rotor misalignment prediction of an induction motor [30]; however, the methodology uses complex and extensive preprocessing steps such as the coefficient of statistical variation, the Boruta algorithm, and the Recursive Feature Elimination (RFE) algorithm. Wang et al. have shown that CNN performs significantly better and reduces preprocessing as compared to SVM [31]. The authors used a three-dimensional STFT feature of the vibration data to classify the imbalanced, broken, and normal rotor. However, it is important to note that the use of 3D signals as input for CNN can be computationally expensive. In [32], the authors focus on a new CNN-based method for detecting various bearing faults of a planetary gearbox. The developed method specializes in cases where datasets are unbalanced or too noisy. The algorithm is promising for data with medium and high signal-to-noise ratios; however, its performance for large-scale industrial data is not known. Zhao et al. (2022) developed a rotor fault diagnostic framework based on the Normalized Conditional Variational Auto-encoder (NCVAE) [33]. The basic theme is to enhance and exploit the feature-learning ability of the NCVAE. Simulation-based bearing and rotor fault data are generated for classification purposes. The model focuses on increasing accuracy when data are imbalanced, as fewer data samples are available for certain classes. The model works well on simulated bearing and rotor fault data; however, as suggested by the authors, the actual industrial data, being complex and noisy, may need a more sophisticated model. All these models use variable depth models as the number of classes and data increases. A single model which can deal with fewer data and fewer classes, as well as multiclass problems and large datasets, has not been optimized for industrial rotor fault applications, which is the focus of this work.

A de facto research trend for prognostic health monitoring (PHM) problems is the use of shallower AI algorithms for binary class prediction with relatively small training data and deeper networks for multiclass high-level data [34]. These algorithms have shown promising results for the specific test conditions; however, shallower networks fail to adapt to complex data in multiclass problems [35], while deep networks degrade in performance on a small number of classes and run into overfitting [36]. This paper proposes a new deep learning algorithm that is deep enough to extract deep features of large datasets used in multiclass problems and also performs well on simple binary classification problems without running into overfitting. The model was developed for a special use case of an industrial rotor problem, using the same data and the same machine learning (ML) model for both binary class and multiclass prediction problems. For training the ML algorithms on vibration data, PHM engineers mostly prefer to use frequency components of signals obtained by Fast Fourier Transform (FFT) [34]. To address this problem, it is a good practice to analyze portions of the signal at various intervals with the help of STFT [37]. The feature-rich STFT has been used in fault diagnosis by several researchers to detect broken rotors [38], faulty bearings [39,40], and motor winding faults [31]. However, it has not been given much attention in the prediction of the unbalance level. FFT is effective for stationary signals but it is not quite powerful enough to analyze non-stationary signals [41]. To exploit the benefits of feature-rich STFT, we utilized STFT as the training input data for the classification of the rotor unbalance. This work focused on developing an online prediction model for multiclass rotor unbalance. When trained with different levels of unbalanced data, the algorithm can be applied to industrial rotors during runtime to predict the type and level of unbalance signaled by the runtime vibration data of the rotor. Hence, the unbalance problems identified in real time are dealt with before escalating into damage and complications. This is of significant help to the operator.

The database used in this study was downloaded from the Fraunhofer Fordatis Research Institute, Germany [42], and was collected on a DC motor setup connected with three vibration sensors. In total, five test condition datasets were recorded; one dataset was measured when there was no unbalance in the rotor, and the remaining four datasets were collected when different levels of unbalance were introduced to the rotor. The main contributions of this work are as follows:

- A new deep learning approach is developed for improved accuracy of binary and multiclass classification. Our model’s basic architecture is derived from ResNet and CNN, and the developed model outperformed both algorithms. In addition, the results are compared with state-of-the-art ML algorithms, which shows the superiority of our algorithm. To the best of our knowledge, this is the first study for the development of this model.
- A core dataset selection strategy is presented to speed up the training process by selecting fewer datasets for training. Among the four datasets for the unbalanced cases, only two were selected for training based on the statistical analysis by observing the standard deviations of the datasets. Two types of classifications were carried out. First, two-class classification for predicting balanced and unbalanced signals was performed (Analysis-1). Afterwards, in Analysis-2, a multiclass classification was performed to categorize the severity of rotor unbalance (refer to Table 1 for details). Analysis-2 is useful to predict the severity of the imbalance in the rotor (divided into four different classes).
- It demonstrates the feasibility of using the STFT feature map for better training, in contrast to conventional FFT as the main feature of data.

The rest of the paper is organized as follows. Section 2 details the system under discussion. Section 3 describes the methodology in detail. An improved deep learning model is proposed in Section 4. Section 5 deals with the results. Finally, the conclusion of this study is given in Section 6.

## 2. System Overview

Rotary machines frequently experience imbalances as a result of high speeds, misalignment, and inappropriate loading or mass deposition. This imbalance is often the starting point of machine failure. One of the frequent causes of mechanical failures is rotor imbalance [8].

### Imbalance in Rotary Machines

**Figure 1:**
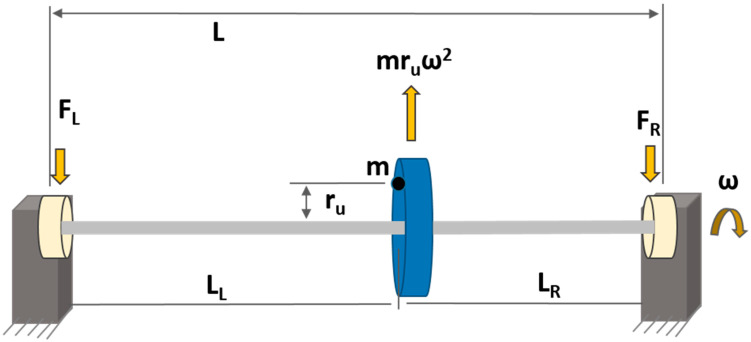
Graphical representation of static imbalance in rotors.

Mathematically, rotor imbalance can be expressed as follows [8]:

(1) $$\vec{U}=m\times {\vec{r}}_{u}$$

where *m* is the added mass in grams and $\vec{r}$*_u_* is the distance in millimeters of added mass from the axis of rotation. The common types of imbalances in rotating machinery are (i) static, (ii) coupled, (iii) quasi-static, and (iv) dynamic [29]. In this article, only static imbalance is considered, which is the most common type of imbalance in industrial machines. Static imbalance is the condition when the center of gravity of a rotor shaft is not aligned with its axis of rotation, primarily due to asymmetric mass distribution [43], as shown in Figure 1.

It is called static because it is always present in rotors, even if they are stationary [43]. Static imbalance produces the centrifugal force induced by mass imbalance when a rotor is in motion, which is stated as follows [44]:

(2) $$F=m\times r_{u}\times \omega^{2}\;$$

where F is the force in Newtons and ω is the speed in radians per second (rad/s). The centrifugal force is balanced by the reaction force on the bearing(s) on the endpoint of the rotor.

## 3. Methodology

### 3.1. System Parameters

**Figure 2:**
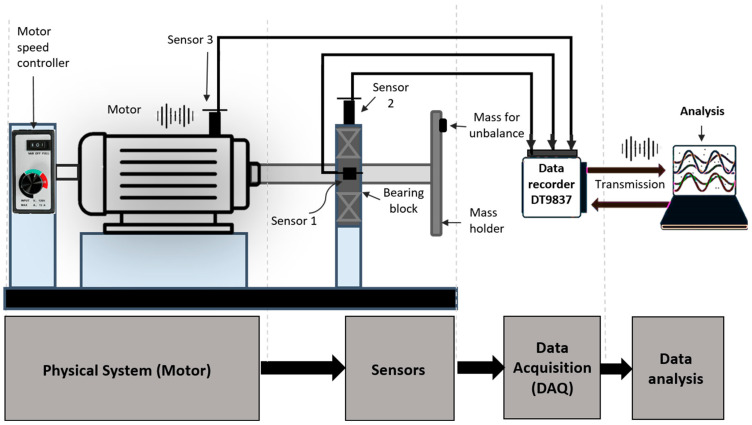
Conceptual design layout of the data acquisition setup.

The data used in this study were collected from a 130-watt DC motor (type UE 511TM, manufactured by WEG GmbH, Kerpen, Germany) [45] connected to a steel shaft 12 mm in diameter and 75 mm in length. The shaft was guided by a roller bearing in a galvanized steel bearing block. The experimental setup is depicted in Figure 2. A 3D-printed bracket for holding the load mass was inserted at the end of the shaft. Unbalance was created by inserting a mass at different radii in the 3D-printed bracket. Three vibration sensors (M001AC) attached to the motor mounting and bearing block were read out using a four-channel data acquisition device (DT9837). The mass (m) and the distance from the axis of rotation (*r_u_*) are the two basic parameters considered for the imbalance factor. Table 1 shows the test conditions used in this study. For each test condition, the motor was swept through a rotation speed between 300 and 2300 revolutions per minute. Motor speed was controlled by a 24 V controller (WEG GmbH, type W2300) mounted on a plate of galvanized steel. A total of 10 datasets were recorded, 5 for training purposes (dataset D) and 5 for evaluation (dataset E). Dataset 0 D,0 E was recorded when there was no unbalanced load acting on the shaft. Datasets 1–4 were recorded when loads acted with various configurations of size and location.

As shown in Equation (1), the product of the mass m and the radius r is a direct measure of the unbalance strength. A large mass and radius will therefore produce the highest unbalance factor (unbalance U4 in our study). Two types of classifications are performed. First, in Analysis-1, a binary classification of balanced (B) and unbalanced (U) is performed, as shown in Table 1. Second, in Analysis-2, a 5-label classification of balance (B) and the level of unbalance (U1, U2, U3, U4) is performed.

### 3.2. Data Selection and Preprocessing

It is important to emphasize that two types of classification analyses (shown in Table 1) are performed in this study. Analysis-2, a multiclass prediction problem, is relatively simple in terms of input data, as for each class label, exactly one dataset is available. Meanwhile, Analysis-1, a binary classification problem—“*Balanced*” and “*Unbalanced*”—has one training dataset D0 for the balanced case and four training datasets (D1–D4) for the unbalanced case. Using all these datasets (D1–D4) in training for the class label “Unbalanced” in Analysis-1 will result in overfitting as well as high computational costs. The large datasets in Analysis-1 will push the model to overfit towards the minute behavior of the data, and hence, a better generalization cannot be achieved. This results in a high computational cost and decreased accuracy. Therefore, only two of the four unbalanced case datasets were used so that the computational advantage could be achieved without compromising the accuracy.

**Figure 3:**
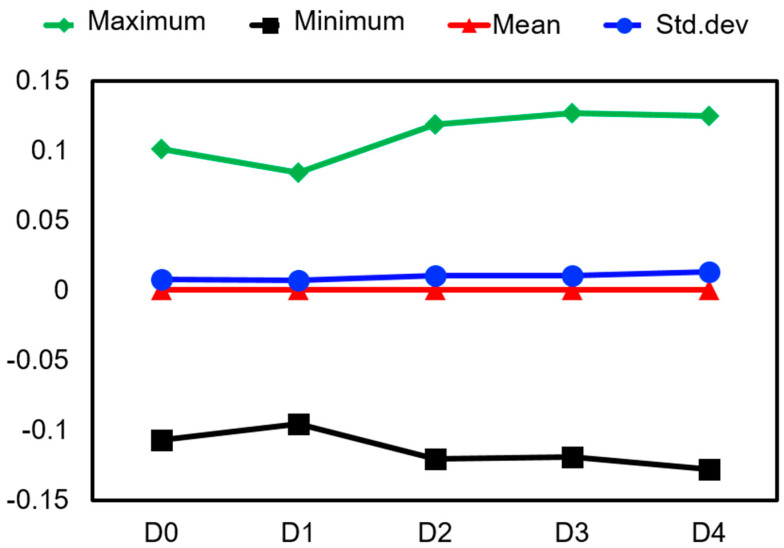
Statistical distribution of the training datasets.

By choosing the core set from huge datasets, it is possible to considerably increase the computational effectiveness of ML models. Even if we omit them from training, the model’s evaluation of D3 and D4 as unbalanced is obvious due to their high data distribution (as shown in Table 2 and Figure 3). On the contrary, D1 and D2 have low standard deviations, similar to the balanced dataset D0. In order for the model to distinguish the unique feature qualities of D1 and D2 from those of D0, it is crucial to choose these datasets for training. This will help the model attain the traits of small local features of data and increase the sensitivity and accuracy of the model. This will also reduce the training time for Analysis-1 and better suit industrial needs when training data are small.

**Figure 4:**
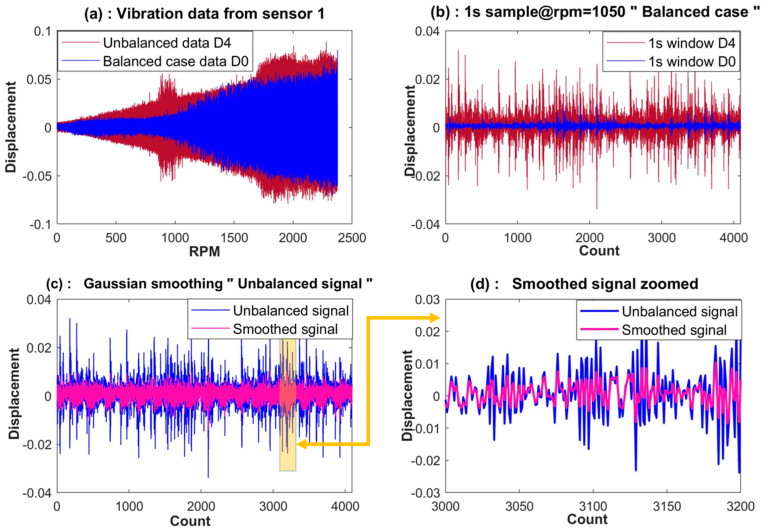
Signal preconditioning: (**a**) balanced dataset D0 and unbalanced dataset D4; (**b**) window sample of D0 and D4; (**c**) smoothing for filtering out noise; (**d**) a portion of the smoothing process zoomed in.

There is always some degree of noise in the signals measured from a real-life mechanical system. Figure 4c shows how the random noise effect is reduced by passing the vibration data through a Gaussian moving average filter. A two-sided moving-average Gaussian filter can be written as follows:

(3) $$y\left(i\right)=\frac{1}{N}\;\overset{+\left(N-1\right)/2}{\underset{j=-\left(N-1\right)/2}{\sum }}w\left(i-j\right)\times x\left(i-j\right)\;$$

where $y\left(i\right)$ is the smoothed value for the ith data point, *N* is the window size, and $w\left(i-j\right)$ are the weights associated to each data point. The filter reduces the noise effect and unwanted data edges in the time domain. A sampling frequency (*Fs*) of 4096 samples per second is used in the data acquisition setup. For simplicity, the filtered raw data are broken into samples of size *Fs*. Each sample is called a window. Each window has 4096 data points. Data from vibration sensor 1, as well as samples of raw and filtered signals, are presented in Figure 4.

### 3.3. Feature Extraction

The filtered data were examined for important features. The most important characteristics of vibration signals are the frequency components. By frequency analysis, it can be seen that there are certain frequencies present in both balanced and unbalanced vibration data that correspond to the operational parameters such as rotation frequency and the number of rotating components such as bearings. Furthermore, in the “Unbalanced case”, additional frequencies are present as well, which are the result of unbalance present in the rotation system. Fourier Transform is an important tool to analyze the characteristic features of a signal.

#### 3.3.1. Fast Fourier Transform

**Figure 5:**
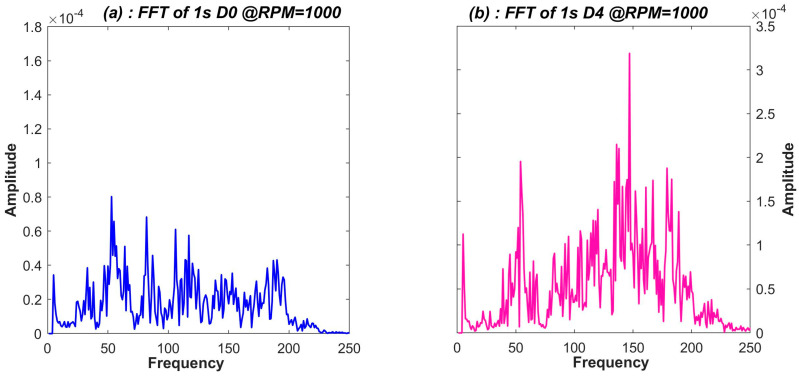
Fast Fourier Transform showing various acting frequencies in (**a**) balanced case, shown in blue, (**b**) unbalanced case, shown in pink.

Fast Fourier Transform (FFT) resolves a signal into its frequency components. FFT gives us acting frequencies hidden in the signal. The unbalance in the machine, due to mass asymmetry, adds extra frequency components and amplitude spikes to the vibration signal. The set of active frequencies and their amplitudes deduced from the FFT signal can be selected as a promising feature for ML algorithms. Figure 5a (blue) and Figure 5b (pink) illustrate sample FFT for balanced and unbalanced case signals, respectively. It is evident from Figure 5b that in the unbalanced case, there are more frequency components and high amplitudes as compared to the balanced case in Figure 5a. Regression-based ML algorithms work well with FFT; however, the efficiency degrades when data complexity and the number of classes increase, as is the case for Analysis-2 (5 classes). For such cases, along with deeper networks, a comprehensive set of features or signal characteristics like STFT can perform better.

#### 3.3.2. Short-Time Fourier Transform

**Figure 6:**
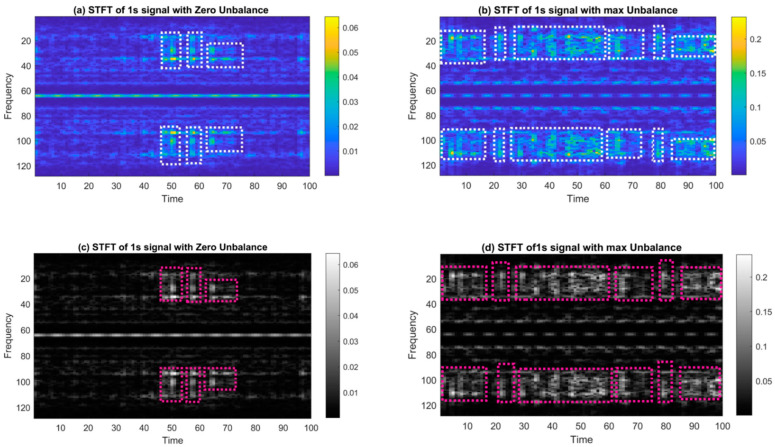
Short-Time Fourier Transform: (**a**) 3D sample for balanced case, (**b**) 3D sample for unbalanced case, active frequency components encircled in white traingles (**c**) grayscale STFT for balanced case, and (**d**) Grayscale STFT for unbalanced case., active frequency components encircled in pink traingles.

Short-Time Fourier Transform (STFT), which is an extension of FFT, allows us to view the frequency characteristics of a signal as a function of time. STFT captures frequency characteristics as a function of time by using sliding windows in time. As a result, a spectrum is formed at discrete time intervals. STFT has better temporal and frequency localization properties compared with Fourier Transform. It is used to generate representations that capture both the local time and frequency content in the signal. When the rotor along with the added mass spins at high speeds, sharp energy gusts inevitably happen in the vibration data. Bright notches, as shown in Figure 6, that change in the time domain are an indication of this effect in the STFT histogram.

Figure 6a is an example of how STFT spectral images were created from signals with a sampling rate of 4096 data points per second. The STFT images are transformed into grayscale 2D images with half the resolution (438 × 328) of the original signal because the STFT images’ high resolution (3D RGB channel; 875 × 656 for one sample) consumes a significant amount of memory and is burdensome for academic-scale machines with memory constraints. This makes the computation process faster. Figure 6a,b display the 3D STFT images for the balanced and unbalanced examples, respectively, whereas Figure 6c,d displays the corresponding grayscale images.

### 3.4. Classification Models

In this work, we focus on the modified ResNet model for our data. For comparison purposes, 5 state-of-the-art classification algorithms are also employed: Artificial Neural Network, Convolution Neural Network, Random Forest, Support Vector Machine, and Xtreme Gradient Boost. These classification techniques are often called algorithm adaptation techniques.

#### 3.4.1. Artificial Neural Network

Artificial Neural Network (ANN) is a machine learning algorithm that mimics the structure and function of the human brain [46]. ANNs are composed of interconnected node layers, containing an input layer, one or more hidden layers, and an output layer. When a node value reaches a certain threshold, the node is activated, and its value is passed on to the next layer of the network. ANNs have been extensively used by many researchers, including [24,47] and others, for fault detection of rotors and bearings.

#### 3.4.2. Random Forest

Random Forest (RF) is a supervised learning algorithm that utilizes ensemble learning techniques to create a robust classifier by combining weaker classifiers [48]. This approach involves training models using a bagging method, which is responsible for the improved performance of the algorithm. As the name suggests, the algorithm builds a “forest” of decision trees, each of which serves as a weak classifier. The output of each decision tree is then merged in parallel to form a strong classifier. Random Forest has been used by many authors [29,42,48] for vibration-based damage classification of rotary machines.

#### 3.4.3. Xtreme Gradient Boost

Xtreme Gradient Boosting (XGBoost) is a decision tree technique based on an ensemble learning algorithm that uses a gradient boosting framework. Its most appealing features are its execution speed and model performance. Many writers [49,50] proved that XGBoost outperforms other ensemble-based approaches such as FFT in terms of vibration characteristics.

#### 3.4.4. Convolutional Neural Network

Convolutional Neural Network (CNN or ConvNets) is a deep learning network architecture that can recognize patterns in input images by assigning importance (learnable weights and biases) to various aspects of images to differentiate one object from another. As CNN learns directly from the data, the data preprocessing for ConvNets is substantially reduced as compared to other classification algorithms. CNNs are frequently employed in the fault detection of bearings [47] and rotors [39].

**Figure 7:**
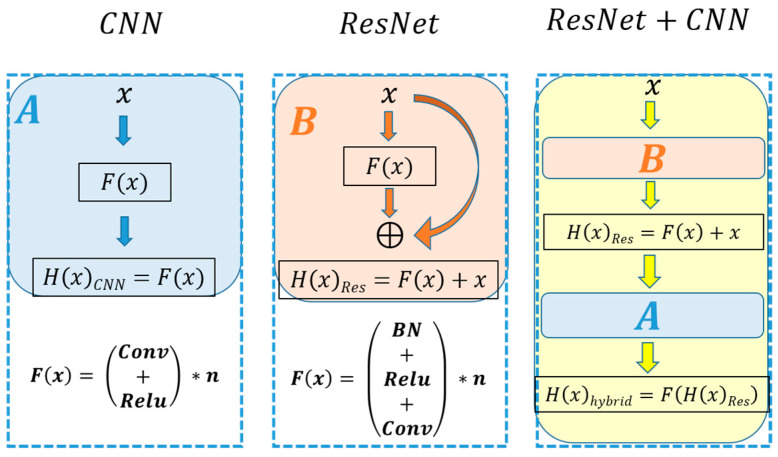
Network architecture comparison: (**left**) conventional CNN, the block funtion (light blue rectangle) is shown as block (**A**), (**middle**) ResNet, the block funtion (light orange rectangle) is shown as block (**B**), and (**right**) proposed hybrid framework, a combination of ResNet and CNN blocks (**B** and **A**).

Convolutional Neural Networks are very effective in extracting important image features in computer vision applications. A typical CNN model is composed of convolutional layers (hidden layers), activation layers, pooling layers, fully connected layers, and a classification layer. When an input feature vector *x* of size *m* × *m* × 1 is passed through a convolutional layer, the convolutional block passes the input through various convolutional filters of size *n* × *n* × 1, which then transforms the input vector into *n* × *n* × 1 convolved feature vectors. The CNN kernels (filters) aim to reduce the number of features present in a dataset by creating a new subset of features that summarizes the overall original set of features. When an input vector x passes through a convolution block, the convolution operations *F*(*x*) are applied to it. The output *H*(*x*), also shown in Figure 7 (left), can be represented as follows:

(4) $$y=H{\left(x\right)}_{CNN}=F\left(x\right)$$

where *H*(*x*) is the desired mapping of the stacked convolutional blocks. It can be deduced from the above *equation* that CNN output is dependent upon the selection of kernels used for the convolution process in the layers. The output mapping *F*(*x*) is passed through a ReLU activation function to classify inputs appropriately, producing a probability from 0 to 1. 

Usually, the presence of more convolutional layers in the CNN model means a better feature vector of the input data (image) can be extracted. However, because of the multiplication of very small gradients, a deep plain neural network also suffers from the vanishing gradient problem or sometimes gradient explosion. The gradient explosion or vanishing problem limits the depth of the network. The ResNet architecture uses a shortcut pass, called a residual connection, to solve the vanishing gradient problem.

#### 3.4.5. ResNet-152

Residual Neural Network (ResNet) is a CNN architecture that makes use of residual connections to overcome the vanishing gradient problem that is typical in deep neural networks [51]. ResNet-152 is an extension of the original ResNet architecture, with a deeper network that has 152 layers. It has been pre-trained on the large-scale ImageNet dataset, which contains over a million images and thousands of categories. This pre-training allows ResNet-152 to be fine-tuned for other image recognition tasks with relatively few training data.

In a residual connection, shown in the middle diagram of Figure 7, the output *H*(*x*)*_Res_* is the sum of the CNN’s output *F*(*x*) and the identity mapping of input *x.* The output *y* of a building block of the residual network is as follows:

(5) $$y=H{\left(x\right)}_{Res}=F\left(x\right)+x\;$$

The formulation in *Equation* (5) can be realized by feedforward neural networks with “shortcut connections”, as shown in Figure 7. Shortcut connections in ResNet serve the purpose of preserving input by skipping one or more layers in the residual block. For normalizing the shifted mean and covariance of the feature maps, batch normalization (BN) is applied before the activation function. Research has shown that among the various deeper versions of ResNet-50/101/152/1202 layers, the 152-layer ResNet offers both depth and high accuracy for better classification. Hence, this research adopted ResNet-152 architecture as the base model of choice.

**Figure 8:**
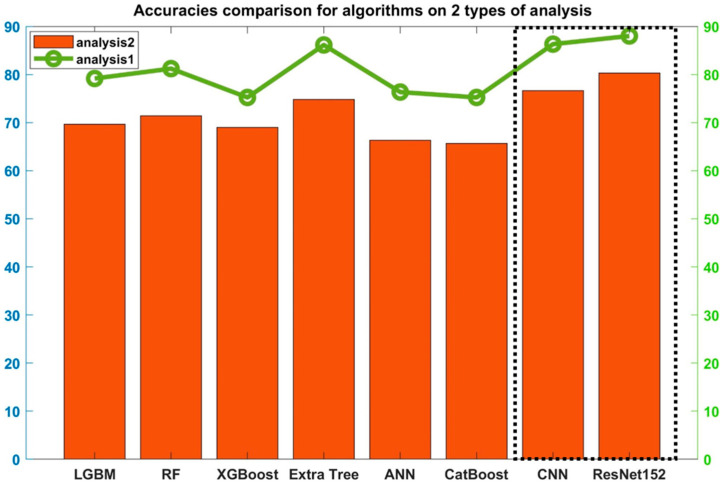
Test accuracies of ResNet CNN and other algorithms on the test data.

In the first stage of our investigation for the model selection, the above-mentioned networks were trained with numerical FFT feature data and image-based STFT data. The two best-performing networks were picked to continue with the development of a robust framework. Figure 8 shows that CNN and ResNet-152 (trained with STFT) outperform the other supervised learning models in terms of accuracy. This highlights the fact that STFT is a desirable feature for our classification problems of Analysis-1 and Analysis-2.

Almost similar to Extra Tree, CatBoost, and RF in terms of accuracy, it can be seen that CNN and ResNet-152 outperform all the algorithms in Figure 8. Both CNN and ResNet have their unique advantages, and it can be hypothesized that a hybrid model with the residual properties of ResNet and the classification accuracy of CNN will perform better than ResNet or CNN alone. A hybrid algorithm with base model ResNet (represented as B in Figure 7) and classification model CNN (represented as A in Figure 7) will have the advantage of depth (of ResNet) for better feature extraction and the advantage of promising classifiers (of CNN). In the following section, a hybrid version of ResNet-152 and CNN for this purpose is described.

## 4. Proposed Framework

We aimed to develop a neural network that is deep enough to successfully extract key features from complex vibration data in both binary and multiclass scenarios. The problem with deep networks is that they often fall victim to overfitting due to the vanishing gradient problem. ResNet is one of the promising deep neural networks that, by using skip connections, can successfully overcome the vanishing gradient problem. ResNet-152 was chosen as the base model of our architecture because of its depth and ability to perform better on complicated multiclass data than other versions of ResNet. This enables the framework to use the feature extraction capability of very deep neural networks without encountering the vanishing gradient problem. ResNet’s skip connections allow information to bypass one or more layers in the network, which can help reduce the vanishing gradient problem and improve gradient flow during training. However, if too many skip connections are employed, the network may struggle to learn increasingly complicated data, and the model may suffer from difficulties such as exploding gradients or overfitting, as evident in Figure 11a. The addition of plain neural network layers will eliminate the problem of exploding gradients while also assisting in the extraction of complex data features. The KERAS library was employed to determine the optimum number of plain neural network layers. Our optimized model has 3 neural layers on top of the 152 residual layers of ResNet. Output values from neural network layers are passed onto the flattened layer, which serves as a horizontal simplified representation of the neuron values. The flattened layer is followed by the fully connected or dense layer, which processes all the information and returns only a few values to determine feature-related values in the image. This output is then condensed in the next step according to the number of output classes and passed to the sigmoid layer for the binary class problem and to the softmax layer for the five-class problem.

**Figure 9:**
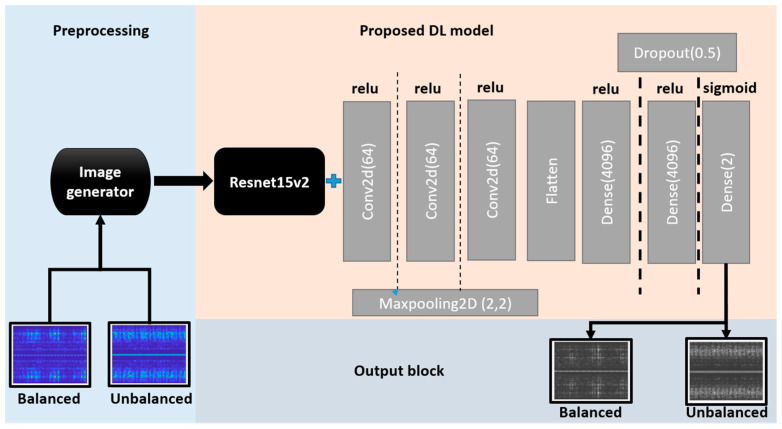
Proposed deep learning framework.

Figure 9 depicts the proposed deep learning framework. Using an image generator, the training data comprising 3D STFT images are resized to 3 × 224 × 224, which is the ResNet input image size. The final layer of ResNet, that is, the fully connected (FC) layer, is removed, and plain convolutional network layers are added.

The categorical cross-entropy loss function *equation* employed for the multiclass problem is denoted as follows:

(6) $$L=-\frac{1}{N}\overset{N}{\underset{i=1}{\sum }}\overset{M}{\underset{j=1}{\sum }}y_{ij}\times \log\left(p_{ij}\right)\;$$

where *N* is the number of training samples, M is the number of classes, *y_ij_* represents the true label of the *ith* sample for the *jth* class (1 if the sample belongs to the class, else 0), and *p_ij_* is the predicted probability of the *ith* sample belonging to the *jth* class. For the binary classification problem, the number of categories is *M* = 2.

To ensure that forward-propagated signals have non-zero variances and hence avoid becoming trivial, batch normalization is performed after each convolution and before the activation function. The Adam optimizer was used for the optimization of the loss function. A 50% dropout rate was selected to avoid overfitting. The k-fold (k = 5) training strategy was used to enable the model to learn and test the characteristics of each dataset by dividing it into five subfolders during training. The key training parameters are given in Table 3.

**Figure 10:**
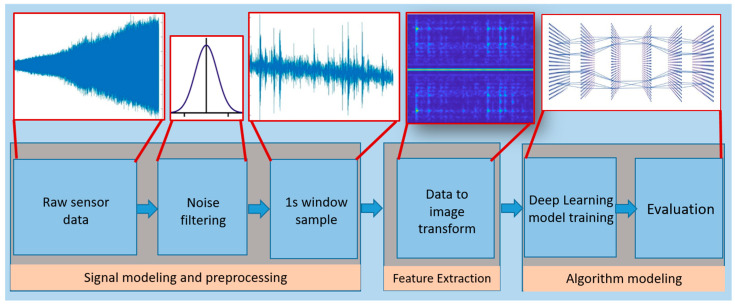
Proposed methodology.

Before the vibration data are sent to the network, they are preprocessed for noise filtering and sampling, followed by extraction of the STFT features. The complete process is shown in Figure 10.

## 5. Results

**Figure 11:**
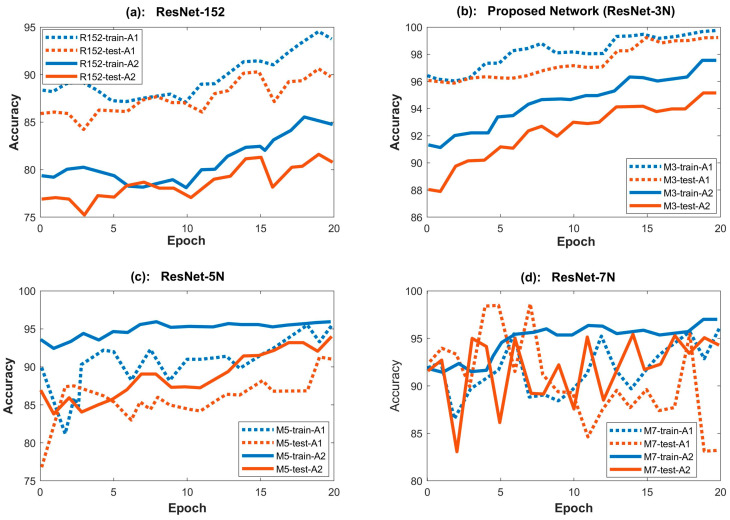
Training accuracies (blue) and test accuracies (orange) for Analysis-1 (solid lines) and Analysis-2 (dotted lines) of (**a**) ResNet-152, (**b**) ResNet-3N, (**c**) ResNet-5N, and (**d**) ResNet-7N.

The proposed framework, trained with the STFT feature data, shows that our network performed best with three neural layers (ResNet-3N). Other neural network combinations with no added neural layers like ResNet-152 or more neural layers like ResNet-5N (with added layers) or ResNet-7N (with seven neural layers) suffer from the overfitting problem either for Analysis-1 or Analysis-2. Accuracy graphs for both Analysis-1 and Analysis-2 are plotted in Figure 11.

The Analysis-1 results (dotted lines) show that network accuracy decreases as the number of neural network (NN) layers increases beyond three. The difference between test and training accuracy increases with five NN layers, and going further, the network suffers from overfitting in the case of ResNet-7N (Figure 11d). The F1-score in Table 4 also follows a similar trend. This suggests that increasing the network depth beyond three NN layers pushes the model to generalize more toward the training data. This behavior is inevitable when the network is deep enough to memorize all data patterns, including noise and random fluctuations. The three NN layers were found to be optimal and could indicate a better way to extract high-, medium-, and low-level features.

In the case of Analysis-2 (solid lines), it is observed that as more NN layers are added to the ResNet-152 architecture, the model behavior is considerably stable and has increased accuracy and a higher F1-score. This is because the problem complexity increases with the increase in data classes (five classes). Consequently, the deeper network is better at identifying key features of each class without suffering from overfitting. However, going for a deeper network is useless as the network’s accuracy does not increase further than that of ReNet-3N for Analysis-2 and will overfit for Analysis-1.

The proposed model gives an increased accuracy of 11.22% for Analysis-1 and 14.84% for Analysis-2 when compared with the original ResNet-152. This is a significant increase.

When the accuracy is compared with CNN, there is a significant increase of 12.92% for Analysis-1 and 18.5% for Analysis-2. These results are given in Table 5. 

Since our proposed hybrid model is better than individual CNN and ResNet-152 for industrial-scale rotor problems, the model is usable for multiclass prediction where a single model can be used for a varying number of classes.

## 6. Conclusions

**Figure 12:**
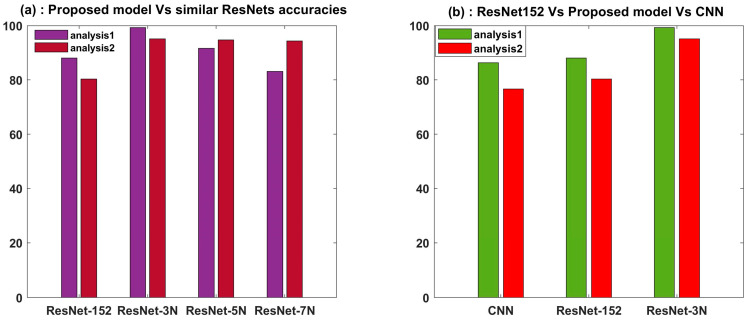
Accuracy comparison of the proposed model architecture (**a**) with added similar experimental designs and (**b**) with the base architectures. It is evidennt that framework ResNet-3N outperforms other similar designs in (**a**) as well as the parent architectures in (**b**).

This paper proposes a robust framework for the classification of balanced and unbalanced vibration signals of a rotor. For the given vibration data, the proposed model displays significantly improved performance in terms of accuracy and F1-score in comparison to other state-of-the-art algorithms used in this field. Other close versions of the model, namely, ResNet-5N and ResNet-7N, performed satisfactorily in Analysis-2 but poorly in Analysis-1, whereas the proposed ResNet-3N performed excellently in both Analysis-1 and Analysis-2. It is concluded that for a multiclass rotor unbalance problem where the number of classes may vary from binary to multiple, ResNet-3N is a robust algorithm that will give high accuracy for industrial rotary machine data. Figure 12 implies that ResNet-3N outperforms similar experimental designs as well as parent architectures of CNN and ResNet-152.

The presented work also demonstrates a strategy to effectively select the core set of training data (from a pool of databases) by evaluating the statistical features of the datasets, which consequently speeds up the training process and increases accuracy. Also, it is deduced that similar to other applications, for rotor unbalance detection and quantification, a rich data representation scheme (such as STFT) performs well in comparison to single-feature FFT data. The STFT histogram (as shown in Figure 6) shows the effect of added mass in the unbalanced rotor in the form of varying frequencies in the time domain. The effect is prominent in transient state data, however is also visible if the rotor is in the steady-state operation. This effect is represented by yellow blotches indicative of energy jumps of specific frequency components. This feature gives intrinsic properties to the STFT and helps deep neural networks characterize the signal with distinguishable properties, making STFT a more promising feature for our deep network.

Computational costs incurred during preprocessing can be a challenge to the real-time application of the method in industry. As image-processing vibration data for STFT feature maps need computational power (processor) and memory (RAM), a computer needs to be attached to the rotor machine for the real-time processing of the data (as shown in Figure 2). The grayscale STFT images can then be sent to a pre-programmed and pre-trained microchip for fault prediction.

For future work, it is possible to test other types of rotor faults on the proposed algorithm. Also, a combination of other data features, like power spectral density, voltage or current values at different faults, rotational speeds, etc., can be tested to see the effect on accuracy. Furthermore, a user interface strategy to transition between binary and multiclass models can be developed for industrial-scale applications in which, with the touch of a button, a user would be able to select the algorithm type for the number of classes to train.

## Figures and Tables

**Table 1:** Parameters of the dataset used. Data classes and datasets vary according to the unbalance factor present in the rotor.

Training Dataset	Evaluation Dataset	Attached Mass (g)	Radius (mm)	Unbalance Factor (mmg)	Analysis-1	Analysis-2
					Class (Total 2)	Class (Total 5)
D0	E0	0	0	0	Balanced (B)	Balanced (B)
D1	E1	3.281 ± 0.003	14 ± 0.1	45.9 ± 1.4	Unbalanced (U)	Unbalanced-1 (U1)
D2	E2	3.281 ± 0.003	18.5 ± 0.1	60.7 ± 1.9		Unbalanced-2 (U2)
D3	E3	3.281 ± 0.003	23 ± 0.1	75.5 ± 2.3		Unbalanced-3 (U3)
D4	E4	6.661 ± 0.007	23 ± 0.1	152.1 ± 2.3		Unbalanced-4 (U4)

**Table 2:** Mean and standard deviation of the training datasets. Distributions of D0, D1, and D2 do not vary much as compared to D3 and D4.

Training Dataset	Minimum	Maximum	Mean	Std. Deviation
D0	−0.10675	0.101037	0.000664	0.00838
D1	−0.09483	0.084269	0.000715	0.007499
D2	−0.11995	0.118957	0.000571	0.010673
D3	−0.1188	0.127205	0.000689	0.010989
D4	−0.12719	0.125183	0.000684	0.013755

**Table 3:** Hyperparameters of the proposed network.

Epoch	Resize	Folds	Patience	Mode	Optimizer	Rate	Beta_1	Beta_2	Activation Function	
									For 2 Class	For 5 Class
20	224,224,3	5	10	max	Adam	0.0001	0.9	0.999	Sigmoid	Softmax

**Table 4:** Test accuracies vs. F1-scores of the tested models. Note that the accuracy and F1-score of the proposed model are the highest.

Model	Accuracy		F1-Score	
	Analysis-1	Analysis-2	Analysis-1	Analysis-2
ResNet-152	88.01	80.31	0.8809	0.8405
ResNet-152-3N	98.71	94.15	0.9759	0.958
ResNet-152-5N	91.59	94.73	0.742	0.8847
ResNet-152-7N	83.2	94.540	0.845	0.910

**Table 5:** The proposed hybrid model has higher accuracy than CNN or ResNet alone. Percent increase is in the last row.

	CNN		ResNet-152		Proposed Model (ResNet-3N)	
	Analysis-1	Analysis-2	Analysis-1	Analysis-2	Analysis-1	Analysis-2
Accuracy	86.31	76.65	88.01	80.31	99.23	95.15
Percent increase (%)	12.92	18.5	11.22	14.84	n/a	n/a

## Data Availability

The datasets in this study were dowloaded form Fraunhoffer research institute and can be found at https://fordatis.fraunhofer.de/handle/fordatis/151.2 (accessed on 9 August 2023).
